# Potential value of urine lateral-flow lipoarabinomannan (LAM) test for diagnosing tuberculosis among severely acute malnourished children

**DOI:** 10.1371/journal.pone.0250933

**Published:** 2021-05-05

**Authors:** Birgit Schramm, Rodrigue C. Nganaboy, Piex Uwiragiye, Didier Mukeba, Aboubacar Abdoubara, Illa Abdou, Jean-Claude Nshimiymana, Seyni Sounna, Laurent Hiffler, Laurence Flevaud, Helena Huerga

**Affiliations:** 1 Epicentre, Paris, France; 2 Médecins Sans Frontières, Niger; 3 Médecins Sans Frontières, Barcelona, Spain; 4 Ministry of Health, Niger; 5 Médecins Sans Frontières, Dakar, Senegal; The University of Georgia, UNITED STATES

## Abstract

**Background:**

Tuberculosis (TB) is a serious co-morbidity among children with severe acute malnutrition (SAM) and TB diagnosis remains particularly challenging in the very young. We explored whether, in a low HIV-prevalence setting, the detection of mycobacterial lipoarabinomannan (LAM) antigen in urine may assist TB diagnosis in SAM children, a pediatric population currently not included in LAM-testing recommendations. To that end, we assessed LAM test-positivity among SAM children with and without signs or symptoms of TB.

**Methods:**

A cross-sectional assessment (February 2016-August 2017) included children <5 years with SAM from an Intensive-Therapeutic-Feeding-Centre in Madaoua, Niger. Group 1: children with signs or symptoms suggestive of TB. Group 2: children without any sign or symptom of TB. Urine-specimens were subjected to Determine^TM^ TB-LAM lateral-flow-test (using a 4-grade intensity scale for positives). LAM-results were used for study purposes and not for patient management. Programmatic TB-diagnosis was primarily based on patients’ clinical symptoms and TB contact history with no systematic access to X-ray or microbiological reference testing.

**Results:**

102 (Group 1) and 100 children (Group 2) were included (median age 18 months, 59.4% male, 1.0% HIV-positive). In Group 1, 22 (21.6%) children were started on TB-treatment (probable TB) and none of the children in Group 2. LAM-positivity was 52.0% (53/102) and 37.0% (37/100) in Group 1 and 2, respectively. Low-intensity (Grade 1) LAM test-positivity was similarly high in both Groups (37.3% and 36.0%, respectively), while Grade 2 or 3-positives were mainly detected in Group 1 (Group 1: 14.7%, Group 2: 1.0%, p<0.001). When considering only Grades >1 as positive, LAM-testing detected 22.7% (95%CI: 7.8, 45.4) among probable TB cases, while 99% (95%CI: 94.6, 99.9) of unlikely TB cases (Group 2) tested negative.

**Conclusion:**

These findings suggest the potential utility of LAM urine testing in HIV-negative children with SAM. Determine LAM-positivity with Grades >1 may identify HIV-negative SAM children that are eligible for rapid TB-treatment initiation, though low-intensity (Grade 1) LAM-positive results may not be helpful in this way. Further studies in this specific pediatric population are warranted, including evaluations of new generation LAM tests.

## Introduction

Tuberculosis (TB) is a frequent co-morbidity among children with severe acute malnutrition (SAM) in TB endemic settings [1,2]. Children with SAM typically present with malnutrition-associated immune-suppression and are more prone to severe forms of TB [3–6]. The mortality risk among patients with TB is also significantly increased with moderate or severe malnutrition [4,7,8]. Better integration of TB diagnosis into malnutrition wards is urgently needed [2,5,6,9].

TB diagnosis is particularly challenging among young children. Clinical symptoms alone are insufficiently accurate for TB case detection [10], with SAM and TB symptoms overlapping. Children can often not produce sputum, and even if nasopharyngeal aspiration or gastric washing is used, the often paucibacillary nature of specimens limits case-identification by smear microscopy, Xpert MTB/RIF or TB culture [11–15]. Chest radiography is not always accessible, and image-interpretation is difficult, especially in younger children. Since 2013, a point-of-care lateral flow (LF) assay that detects the lipoarabinomannan (LAM) antigen of *Mycobacterium* tuberculosis (MTB) in urine specimens (Alere Determine^TM^ TB LAM Ag test, Abbott) has been available. Urine specimens are easy to collect in children and lateral flow tests allow clinicians to obtain results at the point-of-care within 30 minutes. LAM-performance is better among HIV-positive individuals, especially the severely immune-compromised, and TB-LAM LF-testing is currently exclusively recommended to assist TB diagnosis among HIV-seropositive adults and children [16]. Importantly, evidence for LAM-testing in pediatric populations remains scarce. Findings from available studies vary considerably [16–20] and almost no data are available on LAM’s diagnostic value in children with severe malnutrition [21]. Since LAM-test performance is better in immuno-compromised HIV-positive individuals, we hypothesized that it may also have a diagnostic value in (HIV-negative) SAM children since SAM has been linked with immune dysfunction [22–24]. Hence, we assessed LAM test-positivity in hospitalized children <5 years with SAM, with and without TB symptoms, in a low prevalence HIV context in the pediatric intensive feeding center (CRENI) in Madaoua district, Niger. Malnutrition is a major threat to child health in Niger, with an estimated 15% of children severely malnourished in 2018 [25], and TB is endemic in the country with an overall estimate of 84 (54–120) TB cases per 100 000 inhabitants in 2019 [26]. This proof-of-concept study attempts to gain insight into whether TB LAM urine-strip testing may have a role in TB diagnosis in this particularly vulnerable pediatric patient group and provide guidance whether further diagnostic performance evaluations would be of value.

## Materials and methods

### Study design, study site and population

A cross-sectional study was conducted in the Médecins sans Frontières (MSF)-supported Intensive therapeutic in-patient feeding center (CRENI) in Madaoua, Tahoua Region, Niger. The main objective was to assess LAM test-positivity among children hospitalized with SAM and signs or symptoms suggestive of TB. Two groups of children were included. Eligibility criteria (Group 1): <5 years old, hospitalized with diagnosis of SAM, not receiving TB-treatment, and presenting with at least one sign or symptom suggestive of TB at admission or during hospitalization. Based on programmatic data, we expected that 25% of SAM children hospitalized with signs or symptoms suggestive of TB would be diagnosed with TB. We hypothesized that 30% of them would be LAM-positive and 97% of non-TB cases would be LAM-negative. A sample size of 100 children was set to allow us to report 10% overall TB-LAM positivity with a precision of 4.9–17.6% (exact Clopper Pearson 95% confidence interval for proportions, PASS sample size software V13). To support results interpretation in Group 1 (following interim analysis), we included in a second step children (N = 100, Group 2) meeting the following criteria: <5 years old, not on TB treatment, hospitalized *without* any defined TB sign or symptom, no cough, no fever or recent history of fever, responding correctly to nutritional treatment, and not diagnosed with pneumonia. Children hospitalized at the CRENI were consecutively screened for eligibility to the respective study groups, and those meeting the study criteria were included until the sample size was reached. All children were programmatically screened for HIV (two sequential rapid tests followed by confirmatory PCR for infants <12 months if rapid test positive). Follow-up was continued until hospital discharge.

### Definitions

SAM was defined as weight-for-height Z-score <−3 and/or mid-upper arm circumference [MUAC] <11.5 cm, and/or bipedal edemas [27]. Signs and symptoms suggestive of TB were defined as presenting with at least one of the following: a) at admission: contact of a known TB case, persistent cough for >two weeks, unexplained fever for >one week, or suspicion of extra-pulmonary TB; b) during hospitalization: poor weight gain despite appropriate nutritional support, persistent pneumonia after appropriate antibiotic treatment, persistent fever for >1 week after excluding common causes such as malaria or pneumonia, persistent cough, persistent or worsening fatigue, or a chest X-ray suggestive of TB. A diagnosed TB case was defined as a patient initiating TB-treatment. Programmatically, patients were diagnosed with TB by taking their exposure to a TB case into consideration as well as clinical signs of TB and chest X-ray or Xpert MTB- results. TB diagnosis was mainly based on clinical presentation, chest X-ray was not systematically conducted and Xpert tests (using gastric aspiration or nasopharyngeal aspiration) was only conducted in a few children. To estimate the diagnostic yield of Determine-LAM in the absence of a systematic microbiological reference, two categories were identified: “probable TB” cases (Group 1 children diagnosed with TB) and “unlikely TB” cases (Group 2, not diagnosed with TB).

### Urine specimen collection and TB LAM testing

One urine specimen per child was collected with urinary bags (infants) or collection-pots (children who could produce urine on demand). Unprocessed specimens were tested on the same day with Determine-LAM (Alere Determine™ TB LAM Ag test, Abbott Laboratories, Chicago, IL, USA) in the MSF-supported laboratory in Madoua, following manufacturer’s instructions. Four lab technicians were trained to perform the LAM test. Visual result-read out occurred after 25 minutes using the manufacturer-provided reference card displaying 4 band intensities (Grades 1–4, 4 = highest intensity positivity), with all Grades 1–4 reported as “LAM-positive” per manufacturer recommendations. Lab technicians were blinded to participants’ clinical data. Clinicians were also blinded to LAM-results and LAM results were not used for patient management.

### Data collection and statistical analysis

Demographic and clinical data, collected during routine clinical examinations and from patient files, were recorded into study-specific forms. All data, including TB LAM results, were double entered into a password-protected electronic database (REDcap, Research Electronic Data Capture, Vanderbilt, USA) [28]. Descriptive analyses reported medians with interquartile ranges [IQR] or counts with proportions. Pearson chi-squared-, Fisher’s exact tests, or two-sample tests for proportions were performed to assess differences in proportions. Analysis was performed with Stata 15 software (College Station, Texas, USA).

### Ethics

The protocol and protocol amendment (adding Group 2) were approved by the Ministry of Health, Niger, the Comite Consultative National d’ethique, Niger, and the MSF Ethical Review Board. Inclusion of eligible children required a signed informed consent from a caretaker.

## Results

Between February and August 2016, 103 children with SAM and symptoms suggestive of TB were included (Group 1) (N = 104 screened positive for eligibility criteria, one caretaker refused informed consent). One child was excluded (TB LAM test was conducted one day after urine specimen collection, first- and repeat LAM results were “indefinite”) and 102 remained in the final analysis. Between January and August 2017, 100 children hospitalized with SAM but without any symptoms suggestive of TB were included (control Group 2).

### Clinical characteristics, TB diagnosis, and outcomes

**Fig 1:**
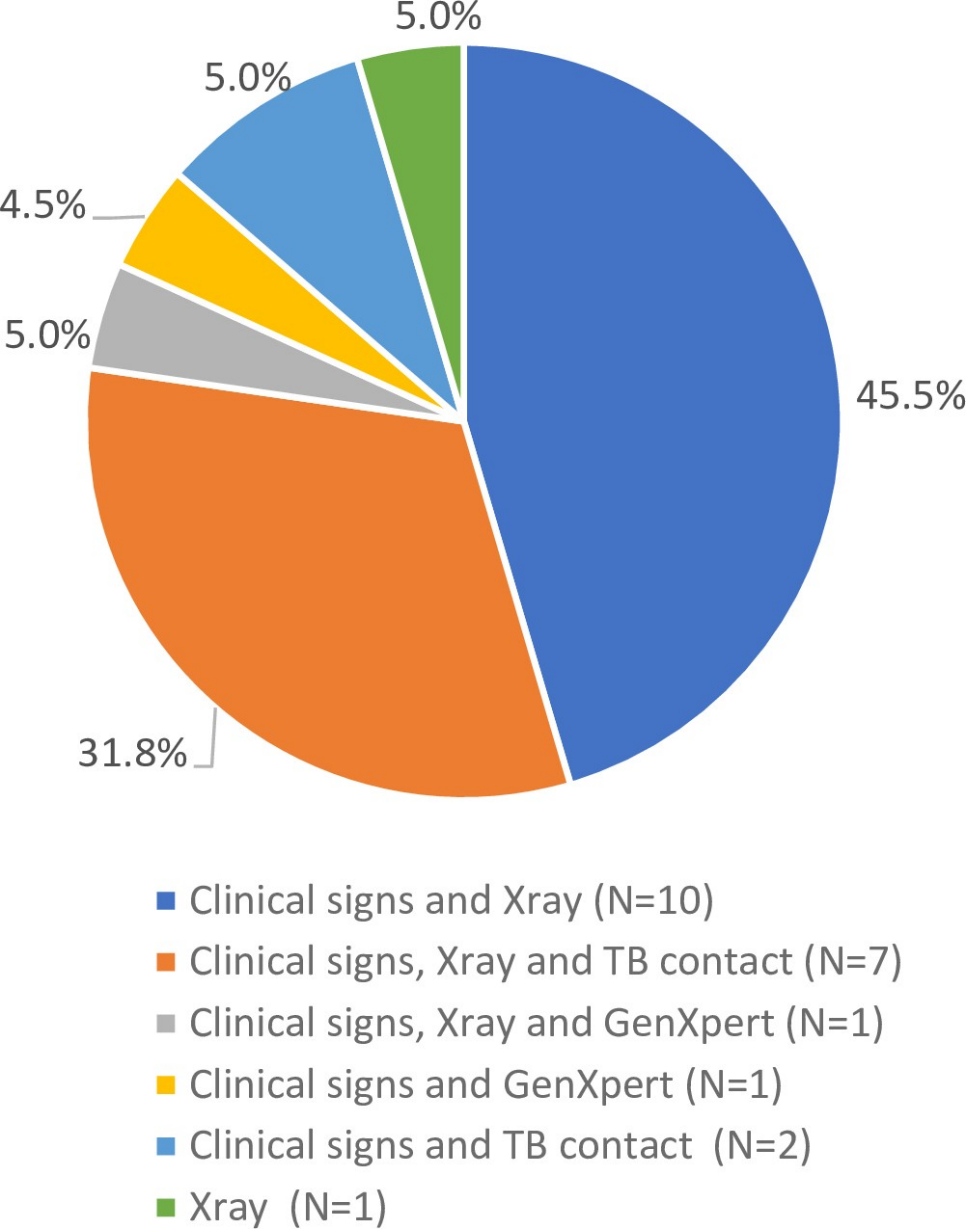
Diagnostic characteristics of 22 children started on TB treatment.

Most children were infants (97% aged <48 months). Two children in Group 1 tested HIV-positive. Children’s demographic and clinical characteristics are shown in Table 1. Most Group 1 children were from intensive care feeding units, while Group 2 children were exclusively from stabilization or pre-discharge units. The symptoms suggestive of TB and results of programmatic TB diagnostic tools in Group 1 are outlined in Table 2. During hospitalization, 42.2% (43/102) of children in Group 1 received a chest X-ray, and 55.8% (24/43) of chest X-rays were interpreted as suggestive of TB. An Xpert-MTB test was performed for seven children (six nasopharyngeal aspirations, one gastric aspiration) and two of them had MTB detected. Overall, 22 children (21.6%) in Group 1 were TB-diagnosed and initiated TB treatment according to the routine standard (none of the 2 HIV-seropositive children did so) (Fig 1). No child in Group 2 (control) was diagnosed with TB. The most frequent diagnoses at hospital-discharge are provided in S1–S3 Tables. Twenty (19.6%) children in Group 1 died during hospitalization (Table 2), two of these were diagnosed with TB.

**Table 1:** Demographic and clinical characteristics at study inclusion.

	Group 1 with signs/symptoms suggestive of TB	Group 2 (control) *without* signs/symptoms suggestive of TB	P
Total, N (%)	102	100	
Female, N (%)	52 (51.0)	30 (30.0)	0.002
Age, months, median [IQR]	19 (11, 24)	15.5 (10, 24)	0.155
Infant (<48 months)	98 (96.1)	98 (98.0)	0.421
HIV-positive per rapid test*	2/100 (2.0)	0	1.0
In-patient unit			<0.001
Admission	1 (1.0)	0	
Stabilization or pre-discharge units	17 (16.7)	100 (100)	
Intensive care unit	81 (79.4)	0	
*missing information*	3 (2.9)	0	
Inclusion, days after admission, median [IQR]	6 (3, 10)	3 (2, 5)	<0.001
Signs of SAM			
Z-score 3 or 4	93 (93.0)	93 (93.0)	0.528
MUAC <115 mm	75 (73.5)	65 (65.0)	0.189
Bilateral oedema	7 (6.9)	12 (12.0)	0.211
Respiratory exam			
Anomalies during auscultation	77 (75.5)	9 (9.0)	<0.001
Tachypnoea	48 (47.1)	1 (1.0)	<0.001
Cyanosis	2 (2.0)	0 (0)	0.498
Hypoxemia (SaO_2_ <90%)	7 (6.9)	0 (0)	0.014
Flapping of the wings of the nose	20 (19.6)	0 (0)	<0.001
Intercostal retraction	62 (60.8)	1 (1.0)	<0.001
Moans and feeds with difficulty	23 (22.6)	0 (0)	<0.001
Fever and increased respiratory rate	49 (48.0)	1 (1.0)	<0.001
Fever and increased heart rate	39 (38.2)	1 (1.0)	<0.001
Weight loss or lack of weight gain	34 (33.3)	3 (3.0)	<0.001
Lymphadenopathy (without fistulae)	6 (5.9)	0 (0)	0.029
Distended abdomen with ascites	4 (4.0)	0 (0)	0.121

*n = 2 HIV-test results missing and n = 1 discordant (unresolved) in Group 1.

**Table 2:** Signs and symptoms of TB at inclusion and TB diagnosis in Group 1.

Total, N (%)	102
Children included with signs or symptoms of TB **at admission**	5 (4.9)
Reported contact of a TB case	2/5 (40.0)
Persistent cough for > 2 weeks	5/5 (100)
Unexplained fever for > 1 week	4/5 (80.0)
Extrapulmonary TB (EPTB) suspected	0
Children included with signs or symptoms of TB **during hospitalization**	97 (95.1)
Reported contact of TB case	13/97 (13.4)
Poor weight gain despite correct nutritional treatment	47/97 (48.5)
Persistence of cough	65/97 (67.0)
Persistent pneumonia after adequate antibiotic therapy	67/97 (69.1)
Persistent fever >1 week after exclusion of malaria or pneumonia	45/97 (46.4)
Persistent or worsening of fatigue	45/97 (46.4)
Signs or symptoms indicative of Extra-Pulmonary Tuberculosis (EPTB)	10/97 (10.3)
Chest X-ray suggestive of TB*	11/97 (11.3)
*Missing information*	5/97 (5.2)
Programmatic diagnosis of TB
Total Chest X-ray performed**	43 (42.2)
Normal	0
Suggestive of TB	24/43 (55.8)
Abnormal/not TB	19/43 (44.2)
Diagnostic specimens collected	6 (6.1)
Sputum	0
Nasopharyngeal aspiration	5/6 (83.3)
Gastric aspiration	1/6 (16.6)
Xpert MTB tests performed	7 (6.9)
MTB detected	2/7 (28.6)
MTB not detected	5/7 (71.4)
Smear Microscopy performed	0
**Initiated TB-treatment**	**22 (21.6)**
Hospitalization outcomes	
Transferred to ambulatory feeding center	81 (79.4)
Died	20 (19.6)
Lost to follow up	1 (1.0)

* N = 11 children had X-ray available at study inclusion (later, N = 31 received an X-ray after inclusion).

** Combined chest-X-ray before or after inclusion.

### TB LAM positivity

Overall LAM-positivity was significantly higher in Group 1 (combination of any Grade 1–4) than in the control Group 2 (52.0% versus 37.0%, p = 0.032) (Table 3). This was mainly due to notably higher proportion of Grades >1 positives in Group 1 (14.7% versus 1.0%, p<0.001). Grade 1 prevalence, however, was similar in both groups (37.3% and 36.0%, respectively). Overall, LAM positivity in Group 1 was not associated with programmatic TB diagnosis (any Grade positive, p = 0.216, S2 Table; Grade>1 positive, p = 0.306, S6 Table).

**Table 3:** TB LAM test results by study group.

	Group 1	Group 2 (control)	
	with signs/symptoms suggestive of TB	without signs/symptoms suggestive of TB	
N (%)	102	100	P*
LAM-negative	49 (48.1)	63 (63.0)	
LAM-positive (any grade)	53 (52.0) (95% CI: 27.9, 47.9)	37 (37.0) (95% CI: 26.6, 46.2)	0.032
Grade 1	38 (37.3)	36 (36.0)	0.854
Grade 2	11 (10.8)	1 (1.0)	
Grade 3	4 (3.9)	0	
Grade 4	0	0	
LAM positive Grade > 1	15 (14.7) (95%CI: 8.5, 23.1)	1 (1.0) (95%CI: 0.003, 5.4)	<0.001

* Two-sample test of proportions.

In Group 1, reported contact with a TB case was significantly associated with (any grade) LAM-positivity (p = 0.025, S2 Table). Among the 20 children who died, 10 (50%) tested LAM-positive (four of these Grade >1) and two had been diagnosed with TB (both Grade 2 LAM-positive). No association between LAM-positivity and in-hospital mortality in Group 1 was found (18.9% of LAM-positives died; 20.4% of LAM-negatives, p = 0.845), and this persisted after considering only Grade >1 as positive (26.7% versus 18.4%, p = 0.456). Most urine specimens (87.1%) were collected with a urine bag. No association was found between LAM-positivity and method of urine collection (not powered, S4 Table).

### Estimation of the diagnostic yield of TB LAM

In a sub-analysis, the diagnostic yield of Grade >1 LAM results was estimated at 22.7% (95%CI: 7.8, 45.4) among the 22 children who initiated TB treatment (‘probable TB cases’). The negative detection rate of TB-LAM (negative or Grade 1) was 99.0% (95%CI: 94.6, 99.9) among the 100 children in Group 2 (‘unlikely TB-cases’) (Table 4). Among the seven children in Group 1 who received an Xpert test, two had MTB-confirmed (one LAM-Grade 1 positive, one LAM-negative). Among the five Xpert-MTB-negative children, three were LAM-positive (Grade 1, 2 or 3, respectively). All three were reported contacts of an active TB case and two began TB treatment (S5 Table).

**Table 4:** TB LAM test results by TB symptoms and/or started on TB-treatment.

	Probable TB (started on TB-treatment in Group 1)	Unlikely TB (Group 2)
	N = 22	N = 100
LAM positive > Grade 1	5	1
LAM-negative or Grade 1 positive	17	99
Positive detection yield	22.7% (95%CI 7.8, 45.4)	-
Negative detection yield	-	99.0% (95%CI 94.6, 99.9)

## Discussion

This proof-of-concept study explored the potential benefit of TB-LAM urine testing for <5 year old children hospitalized with SAM and signs or symptoms suggestive of TB, a population particularly in need of rapid TB diagnosis. Since TB LAM-testing achieves its best performance in immune-compromised HIV-positive patients [29], we postulated that LAM testing may have a diagnostic value in this pediatric population where malnutrition-associated immuno-suppression has been reported [22–24] and who are currently not included in LAM-testing recommendations if HIV-negative [29]. Consistent with the low HIV-prevalence in Niger [30], nearly all SAM-children included in the study were HIV-negative. Our findings indicated that in this group, higher grade (>1) positivity in Determine-LAM results may have identified at least some probable TB cases in SAM children who could have benefitted from fast-track TB treatment initiation. These preliminary findings are encouraging and emphasize that further TB-LAM diagnostic performance evaluations are warranted in SAM children. On the other hand, low-intensity (Grade 1) results may not be helpful to diagnose TB in this population.

Per the manufacturer’s instructions, all Determine-LAM intensity grades (1–4) are reported as positives [31]. While the lowest intensity (Grade 1) positive results were similarly high in both study groups (37.3% and 36.0%), higher grade positive results were about 15-times more frequent in Group 1 than in Group 2 (14.7% versus 1.0%, p<0.001). Although programmatic underdiagnosis of TB is possible in our setting, the strict eligibility criteria for Group 2 (without any signs or symptoms of TB, no cough, and no diagnosed TB), indicate that some LAM-positive Grade 1 results may have been false-positives in our study population. Then again, one of the only two children with a confirmed Xpert MTB diagnosis was also LAM Grade 1 positive, indicating that some Grade 1 children may also be true positives. In other studies, false-positive LAM-urine strip-tests in children have been attributed to the potential cross-reactivity of bacteria from perineal skin, or with stool-contamination during sampling with urine bags (their use may last several hours) [17,32], or to prolonged specimen storage at ambient temperatures [33]. In our study, most included children were infants, and sampling occurred primarily with urine bags (87% of specimen). To address the contamination risk, children were cleaned prior to urine collection, specimens were collected within a short time frame (median 30 minutes), and LAM tests were conducted on fresh specimens (median of 1.8 hours after collection). LAM-positivity was similar for specimens collected with bags or collection cups.

Analysis that considered only LAM-results grades >1 as “LAM-positive” suggested that LAM urine-testing may support the rapid identification of around one in five TB cases in SAM children with signs or symptoms suggestive of TB, with a high negative detection rate (99.0%) among unlikely TB cases (compared to 64% when using any grade as positive). Notably, Nicol and colleagues reported a significant increase in specificity from 66% to 97% (together with a pronounced loss of sensitivity) of Determine-LAM when omitting Grade 1 (out of 4 grades) from “positive” results among children (20% HIV-positive, 25% malnourished) [21]. A multi-site study found high ease-of-use for Determine-LAM operators and very good inter-reader agreement [34]. However, some operators reported difficulties distinguishing “fainter than Grade 1” intensity bands [34]. Although lab technicians in our study were well trained, we cannot fully exclude that difficulties distinguishing Grade 1 from indefinite results may gave existed in our study. Overall, only 2 indefinite results were reported in our data.

To date, only one other study has assessed LAM-performance in SAM children using Determine-LAM (45 children hospitalized in Mozambique, 22% HIV-positive). In this study, among the 17 children (37.5%) clinically diagnosed with TB, all tested Determine-LAM positive but Xpert MTB-negative, suggesting that LAM may have detected TB cases otherwise missed by sputum-based microbiological testing [35]. Similarly, we observed three LAM-positives (Grade 1, 2 and 3, respectively) among the five children with an Xpert MTB-negative result. All three were contacts of an active TB case and two of these initiated TB treatment (both with Xray results suggestive of TB) (S5 Table). The newly developed SILVAMP TB-LAM Assay (“FujiLAM”, Fujifilm) showed superior diagnostic sensitivity over Determine-LAM in HIV-positive adults [36], with highest diagnostic yield among the severely immune-compromised [37]. Among children with presumptive pulmonary TB (20% HIV-positive) [21], both Fuji-LAM and Determine-LAM showed moderate sensitivity (42% and 50%) compared to MTB-culture or Xpert-(Ultra), with substantially higher specificity in Fuji-LAM (92% versus 66%). Importantly, the sensitivity of both FujiLAM and Determine were notably higher among malnourished children (62% vs 31% FujiLAM, 67% vs 46% Determine-LAM) [21]. A second study compared Determine and Fuji-LAM among children with presumptive TB (15% HIV-positive) in four African countries [38]. While Fuji-LAM and Determine had comparable specificity (87.8% and 83.8%), sensitivity was notably higher with Fuji-LAM (64.9% versus 30.7%) using reference standards Xpert Ultra or MTB culture. Interestingly, in this study, Fuji-LAM sensitivity was higher among HIV-negative than HIV-positive children [38]. Together with these recent reports, our findings emphasize the potential value of LAM-testing for diagnosing TB in HIV-negative, SAM children.

Urinary LAM-positivity was also proposed as a prognostic marker of clinical severity and mortality in HIV-positive adults [39–41] or hospitalized HIV-positive children [42]. In our study, most children in Group 1 were from intensive care units. In-hospital mortality was very high in this group (19.6%) (MSF reported a 5.9% overall mortality rate in the facility in 2016; 6.3% in 2017). TB-LAM test-positivity was not associated with in-patient mortality in our sample (not specifically powered).

A limitation of our study is that TB-LAM-performance was explored in a programmatic context with inadequate access to TB diagnostic tools appropriate for children. No systematic microbiological or molecular confirmation of MTB was available. The risk of underdiagnosis (as well as a degree of misclassification) of TB needs to be considered and the LAM-test diagnostic yield may have been underestimated. The study was not specifically powered for the exploratory analyses on the diagnostic yield of LAM-testing. Post-discharge data on delayed TB diagnosis or mortality were not available, also limiting the interpretation of LAM-test performance.

## Conclusions

In this proof-of-concept study, Determine-LAM-test-positivity was frequent in hospitalized SAM-children with and without symptoms suggestive of TB. Determine-LAM-positivity Grade >1 may identify a significant proportion of SAM children eligible for rapid TB-treatment initiation. However, low-intensity (Grade 1) LAM-positive results may not be helpful to diagnose TB in this population. These findings suggest the potential utility of LAM urine testing for TB-diagnosis in severely malnourished, HIV-negative children, a vulnerable population that is currently not included in LAM-testing recommendations. They are encouraging results, and call for researchers to include HIV-negative, severely malnourished children in future diagnostic performance studies, including systematic reference standards and new generation TB-LAM assays that are now becoming available.

## Supporting information

S1 Table Main diagnoses at discharge (if ≥ 5%), by Group. (DOCX)

S2 Table Characteristics of TB-LAM positive and TB-LAM negative, Group 1. (DOCX)

S3 Table If LAM >1 grade positive: main diagnoses at discharge, by Group. (DOCX)

S4 Table Urine specimen collection for TB LAM test (Group 1 and 2 combined). (DOCX)

S5 Table LAM results among N = 7 children tested with MTB-Xpert, Group 1. (DOCX)

S6 Table Programmatic TB diagnosis and TB LAM test results (>grade 1), Group 1. (DOCX)
