# Correction: Potential value of urine lateral-flow lipoarabinomannan (LAM) test for diagnosing tuberculosis among severely acute malnourished children

**DOI:** 10.1371/journal.pone.0256717

**Published:** 2021-08-19

**Authors:** Birgit Schramm, Rodrigue C. Nganaboy, Piex Uwiragiye, Didier Mukeba, Aboubacar Abdoubara, Illa Abdou, Jean-Claude Nshimiymana, Seyni Sounna, Laurent Hiffler, Laurence Flevaud, Helena Huerga

## Notice of Republication

A dataset that was not intended for publication was erroneously included in the originally published article. This article was republished on June 30, 2021 to correct for this error. Please download this article again to view the correct version.

The authors have also provided an updated Data Availability Statement here: Due to the nature of the study and the potential ease of identification of research participants, publication of the data underlying this study is subject to legal and ethical restrictions. The minimal data set underlying the findings of this study are instead available on request, in accordance with the legal framework set forth by Médecins Sans Frontières (MSF) data sharing policy, which ensures that data will be available upon request to interested researchers while addressing all security, legal, and ethical concerns (https://www.msf.org/sites/msf.org/files/msf_data_sharing_policycontact_infoannexes_final.pdf). For data access all readers may contact Robert Nsaibirni, Data Protection and Compliance Officer (robert.nsaibirni@epicentre.msf.org).
