# Supplementary material for: Potential value of urine lateral-flow lipoarabinomannan (LAM) test for diagnosing tuberculosis among severely acute malnourished children
Source: PLoS One. 2021 May 5;16(5):e0250933. doi: 10.1371/journal.pone.0250933 (PMC8099085; doi:10.1371/journal.pone.0250933)
Supplement: S1 Table — (DOCX) [file pone.0250933.s001.docx]

**Table S1:** Main diagnoses at discharge (if ≥ 5%), by Group

| **Group 1 (with signs and symptoms suggestive of TB), N (%)** ^£^ | | N=102 |
| --- | --- | --- |
| Respiratory infection | 762 (74.5) | |
| Anemia | 63 (61.8) | |
| Sepsis | 31 (30/4) | |
| Diarrhea/enteritis ± | 24 (23.5) | |
| Malaria | 21 (20.6) | |
| **Tuberculosis (started on TB treatment)** ^≠^ | **22 (21.6)** | |
| Drepanocytes | 6 (5.9) | |
| Candidiasis | 5 (4.9) | |

| **Group 2 (control), N (%)** ^£^ | N=100 | |
| --- | --- | --- |
| Diarrhea/enteritis ± | 66 (66.0) | |
| Malaria | 40 (40.0) | |
| Anemia | 25 (25.0) | |
| Marasme | 23 (23.0) | |
| Kwashiorkor | 9 (9.0) | |
| Respiratory infection | 7 (7.0) | |
| Dehydration | 7 (7.0) | |
| Candidiasis | 6 (6.0) | |
| ^£^ Non-exclusive listing, combines primary and secondary diagnoses  ^≠^ One child was initially discharged after Malaria diagnosis, re-admitted one week later, diagnosed with TB and started on treatment (chest x-ray suggestive of TB, Xpert test MTB-negative at initial hospitalization).  ± Combined: Diarrhea (most frequently recorded) and few cases of enteritis or dysentery | |  |
