# Supplementary material for: Potential value of urine lateral-flow lipoarabinomannan (LAM) test for diagnosing tuberculosis among severely acute malnourished children
Source: PLoS One. 2021 May 5;16(5):e0250933. doi: 10.1371/journal.pone.0250933 (PMC8099085; doi:10.1371/journal.pone.0250933)
Supplement: S2 Table — (DOCX) [file pone.0250933.s002.docx]

**Table S2:** Characteristics of TB-LAM positive and TB-LAM negative, Group 1

|  | TOTAL |  | LAM-negative | | | | LAM-  positive* | | P ^**^ | |  |  |
| --- | --- | --- | --- | --- | --- | --- | --- | --- | --- | --- | --- | --- |
| Total, N (%) | 102 (100) |  | 49 (100) | | | | 53 (100) | | - | |  |  |
| Age, months, median (IQR) | 19 (11, 24) | |  | | 23 (11, 24) | | 18 (10, 24) | | 0.226 |  |  |  |
| Sex, female | 52 (50.9) |  | | 22 (44.9) | | | 30 (56.6) | | 0.237 | |  |  |
|  |  |  |  | | | |  | |  | |  |  |
| **Signs of SAM recorded at inclusion** |  |  |  | | | |  | |  | |  |  |
| Z-score -3 or -4 | ^#^  97 (95.1) |  | 46 (93.9) | | | | 51 (96.2) | | 0.463 | |  |  |
| MUAC <115 cm | 75 (73.5) |  | 34 (69.4) | | | | 41 (77.4) | | 0.362 | |  |  |
| Bilateral Oedema | 7 (6.9) |  | 3 (6.1) | | | | 4 (7.6) | | 0.776 | |  |  |
| HIV-positive | ^##^ 2/100 (2.0) |  | 1/48 (2.1) | | | | 1/52 (1.9) | | 1.0 | |  |  |
| **Signs or symptoms suggestive of TB**  **at inclusion** |  |  |  | | | |  | |  | |  |  |
| Contact of a TB case | 15 (14.7) |  | 3 (6.1) | | | | 12 (22.6) | | 0.025 | |  |  |
| Persistent cough (combined) | 70 (68.6) |  | 32 (65.3) | | | | 38 (71.7) | | 0.487 | |  |  |
| Unexplained fever (combined) | ^#^ 50 (49.0) |  | 24 (49.0) | | | | 26 (49.1) | | 0.994 | |  |  |
| Persistent pneumonia (after antibiotics) | 70 (68.6) |  | 33 (67.4) | | | | 37 (69.8) | | 0.789 | |  |  |
| Poor weight gain despite nutritional support | 47 (47.0) |  | 26 (55.3) | | | | 21 (44.7) | | 0.168 | |  |  |
| Persistent or worsening fatigue | 47 (46.1) |  | 23 46.9) | | | | 24 (45.3) | | 0.867 | |  |  |
| Suspected extra-pulmonary TB | 10 (9.8) |  | 4 (8.2) | | | | 6 (11.3) | | 0.592 | |  |  |
| **Respiratory exam at inclusion** |  |  |  | | | |  | |  | |  |  |
| Anomalies during auscultation | 77 (75.5) |  | 35 (71.4) | | | | 42 (79.3) | | 0.359 | |  |  |
| Tachypnoea | 48 (47.1) |  | 19 (38.8) | | | | 29 (54.7) | | 0.107 | |  |  |
| Cyanosis | 2 (2.0) |  | 1 (2.0) | | | | 1 (2.09) | | 1.0 | |  |  |
| Hypoxemia (Sa=2 <90%) | 7 (6.9) |  | 3 (6.1) | | | | 4 (7.6) | | 1.0 | |  |  |
| Flapping of the wings of the nose | 20 (19.6) |  | 9 (18.4) | | | | 11 (20.8) | | 0.762 | |  |  |
| Intercostal retraction | 62 (60.8) |  | 29 (59.2) | | | | 33 (62.3) | | 0.750 | |  |  |
| Moans and feeds with difficulty | 23 (22.6) |  | 7 (14.3) | | | | 16 (30.2) | | 0.055 | |  |  |
| Fever and increased respiratory rate | 49 (48.0) |  | 23 (46.9) | | | | 26 (49.1) | | 0.831 | |  |  |
| Fever and increased heart rate | 39 (38.2) |  | 17 (34.7) | | | | 22 (41.5) | | 0.479 | |  |  |
| Weight loss or lack of weight gain | 34 (33.3) |  | 20 (40.8) | | | | 14 (26.4) | | 0.123 | |  |  |
|  |  |  |  | | | |  | |  | |  |  |
| ***Table S2 continued*** | TOTAL |  | LAM-negative | | | | LAM-  positive* | | P ** | |  |  |
| **TB Diagnostics (programmatic)** |  |  |  | | | |  | |  | |  |  |
| Chest X-ray done | 43 (42.2) |  | 18 (36.7) | | | | 25 (47.2) | | 0.286 | |  |  |
| Chest X-ray suggestive of TB | 24/43 (44.2) |  | 8//18 (44.4) | | | | 16/25 (64.0) | | 0.203 | |  |  |
| GeneXpert test done | 7 (6.9)** |  | 3 (6.1) | | | | 4 (7.6) | | 1.0 | |  |  |
| GeneXpert MTB detected | 2/7 (28.6)*** |  | | | | 1/3 (33.3) | | 1/4 (25.0) | 1.0 | | |  |
| **Diagnoses at discharge (if ≥ 5%)** ^£^ |  |  | | | |  | |  |  | | |  |
| Respiratory infection | 76 (74.5) |  | | | | 37 (75.5) | | 39 (73.6) | 0.824 | | |  |
| Anemia | 63 (61.8) |  | | | | 30 (61.2) | | 33 (62.3) | 0.914 | | |  |
| Sepsis | 31 (30.4) |  | | | | 15 (30.6) | | 16 (30.2) | 0.963 | | |  |
| Diarrhea/enteritis ± | 24 (23.5) |  | | | | 11 (22.5) | | 13 (24.5) | 0.805 | | |  |
| Malaria | 21 (20.6) |  | | | | 9 (18.4) | | 12 (22.6) | 0.594 | | |  |
| ***Tuberculosis*** | ***22 (21.6)*** |  | | | | ***8 (16.3)*** | | ***14 (26.4)*** | ***0.216*** | | |  |
| Drepanocytes | 6 (5.8) |  | | | | 4 (8.2) | | 2 (3.8) | 0.342 | | |  |
| Candidiasis | 5 (5.1) |  | | | | 2 (4.4) | | 3 (5.8) | 0.77 | | |  |
| Days of hospitalization, median, (IQR) | 13 (9, 17) |  | | | | 12 (9, 17) | | 13 (9, 18) | 0.598 | | |  |
| **Outcome at discharge** |  |  | | | |  | |  |  | | |  |
| Transferred to ambulatory nutrition center | 81 (79.4) |  | | | | 38 (77.6) | | 42 (80.8) | 0.655 | | |  |
| Died or LTFU ≠ | 21 (20.6) |  | | | | 11 (22.5) | | 10 (18.9) |  | | |  |
| * any Grade (1-4) LAM-positive  ** n=2 of seven with a GeneXpert test were LAM grade 2 or 3 positive, both were GeneXpert-negative  *** n=1 of the 2 MTB-GeneXpert positives was LAM grade 1 positive, n=1 was LAM-negative  # n=1 missing  ## n=1 discordant, n=1 not tested  ** Chi-square test, Fisher exact, or Wilcoxon rank-sum  ^£^ Combined primary and/or secondary diagnosis at discharge, non-exclusive  ± Combined: Diarrhea (most frequently recorded) and few cases of enteritis or dysentery  ≠ n=1 LTFU (lost to follow up) | | | | | | | | | | | | |
