# Supplementary material for: Potential value of urine lateral-flow lipoarabinomannan (LAM) test for diagnosing tuberculosis among severely acute malnourished children
Source: PLoS One. 2021 May 5;16(5):e0250933. doi: 10.1371/journal.pone.0250933 (PMC8099085; doi:10.1371/journal.pone.0250933)
Supplement: S3 Table — (DOCX) [file pone.0250933.s003.docx]

**Table S3:** If LAM >1 grade positive: main diagnoses at discharge, by Group

| **Group 1 (with signs and symptoms suggestive of TB), N (%)** ^£^ | | **N=15** |  |
| --- | --- | --- | --- |
| Respiratory infection^≠^ | 10 (66.7) | | |
| Anemia | 10 (66.7) | | |
| ***Tuberculosis (started on TB treatment)*** | 5 (33.3) | | |
| Sepsis | 4 (26.7) | | |
| Diarrhea/enteritis ± | 3 (20.0) | | |
| Malaria | 3 (20.0) | | |
| Hypovolemic shock & intoxication with traditional product | 1 (6.7) | | |
|  |  | | |
| **Group 2 (control), N (%)** ^£^ | **N=1** | | |
| Diarrhea | 1 (100) | | |

^£^ Combined primary and/or secondary diagnosis at discharge, non-exclusive

± Combined: Diarrhea (most frequently recorded) and few cases of enteritis or dysentery
