# Supplementary material for: Potential value of urine lateral-flow lipoarabinomannan (LAM) test for diagnosing tuberculosis among severely acute malnourished children
Source: PLoS One. 2021 May 5;16(5):e0250933. doi: 10.1371/journal.pone.0250933 (PMC8099085; doi:10.1371/journal.pone.0250933)
Supplement: S4 Table — (DOCX) [file pone.0250933.s004.docx]

## **Table S4:** Urine specimen collection for TB LAM test (Group 1 and 2 combined)

|  | | LAM-negative  N=112 (100) | | LAM-positive*  N=90 (100) | | P ** | |
| --- | --- | --- | --- | --- | --- | --- | --- |
| Urine collection, N (%) | |  | |  | |  | |
| - with a urine bag | | 101 (90.2) | | 75 (83.2) | | 0.276 | |
| - with a collection cup | | 10 (8.9) | | 12 (13.3) | |  |  |
| - with a urine catheter | | 1 (0.9) | | 3 (3.3) | |  |  |
| Urine quality, N (%) | |  | |  | |  | |
| - normal | | 112 (100) | | 90 (100) | |  | |
| - hemorrhagic | | 0 | | 0 | |  | |
| Overall duration of urine collection, minutes, median [IQR] | | 30 [15, 41] | | 30 [15, 40] | | 0.845 | |
| - duration with a bag, minutes | | 30 [15, 40] | | 30 [18, 45] | | 0.744 | |
| - duration with a collection cup | | 12 [3, 34] | | 12.5 [0.5, 32] | | 0.842 | |
| - duration with a catheter | 48 [*na*] | | 17 [10, 30] | | NA | |  |
| Time between urine collection and TB LAM test,  Hours, median [IQR] | | 1.8 [1.2, 2.5] | | 1.6 [1.1, 2.5] | | 0.864 | |
| Duration of LAM-test until reading of result,  Minutes, median [IQR] | | 25 [25, 25] | | 25 (25, 25] | | 0.752 | |

*any grade (Grade 1-4) positive, **Chi-square test, Fisher exact or Wilcoxon rank-sum test
