# Supplementary material for: Potential value of urine lateral-flow lipoarabinomannan (LAM) test for diagnosing tuberculosis among severely acute malnourished children
Source: PLoS One. 2021 May 5;16(5):e0250933. doi: 10.1371/journal.pone.0250933 (PMC8099085; doi:10.1371/journal.pone.0250933)
Supplement: S5 Table — (DOCX) [file pone.0250933.s005.docx]

**Table S5:** LAM results among N=7 children tested with MTB-Xpert, Group 1

| **Seq No** | LAM-result | LAM grade | Started on TB treatment (probable TB case) | Reported TB contact case | X-ray done | X-ray interpretation | Xpert result |
| --- | --- | --- | --- | --- | --- | --- | --- |
| 1 | **positive** | **1** | yes | yes | yes | suggestive TB | MTB not detected |
| 2 | **positive** | **2** | no | yes | yes | anormal/not TB | MTB not detected |
| 3 | negative | na | yes | no | yes | suggestive TB | MTB not detected |
| 4 | negative | na | no | no | yes | anormal/not TB | MTB not detected |
| 5 | **positive** | **3** | yes | yes | yes | suggestive TB | MTB not detected |
| 6 | **positive** | **1** | yes | no | yes | suggestive TB | **MTB confirmed** |
| 7 | negative | na | yes | no | yes | anormal/not TB | **MTB confirmed** |

Legend: Among seven children in group 1 with an Xpert test, two had MTB-confirmed (one was LAM-grade 1 positive, one LAM-negative). Among the five Xpert-MTB-negative children, three were LAM-positive (grade 1, 2 or 3, respectively). All three were reported contacts of an active TB case and two of these were started on TB treatment by the program (both with Xray suggestive of TB (see table S5).
