# Supplementary material for: Potential value of urine lateral-flow lipoarabinomannan (LAM) test for diagnosing tuberculosis among severely acute malnourished children
Source: PLoS One. 2021 May 5;16(5):e0250933. doi: 10.1371/journal.pone.0250933 (PMC8099085; doi:10.1371/journal.pone.0250933)
Supplement: S6 Table — (DOCX) [file pone.0250933.s006.docx]

**Table S6:** Programmatic TB diagnosis and TB LAM test results (>grade 1), Group 1

|  | LAM negative or Grade1 | LAM > Grade 1 |  |
| --- | --- | --- | --- |
| N (%) | 87 (100) | 15 (100) | p |
| Not started on TB treatment | 70 (80.5) | 10 (66.7) | 0.306 |
| Started on TB treatment | 17 (19.5) | 15 (33.3) |  |

P= Fisher’s exact test
